# Bis[*N*,*N*′-bis­[(1*H*-pyrrol-2-yl)methyl­ene]cyclo­hexane-1,2-diamine]titanium(IV) tetra­hydro­furan solvate

**DOI:** 10.1107/S1600536808002870

**Published:** 2008-01-30

**Authors:** Xue-Qin Zhang, Bin Xu, Ya-Hong Li, Wu Li

**Affiliations:** a Department of Chemistry and Chemical Engineering, Suzhou University, Suzhou 215123, People’s Republic of China; b Institute of Salt Lakes, Chinese Academy of Sciences, Xining City, Qinghai Province 810008, People’s Republic of China

## Abstract

In the title compound, [Ti(C_16_H_18_N_4_)_2_]·C_4_H_8_O, the Ti^IV^ ion is chelated by two Schiff base dianions with a TiN_8_ distorted square-anti­prismatic coordination geometry. The two cyclo­hexane rings assume the typical chair conformation. No hydrogen bonding is observed in the crystal structure.

## Related literature

For general background, see: Li *et al.* (2002 ▶); Gardner *et al.* (2001 ▶); Han *et al.* (2007 ▶).
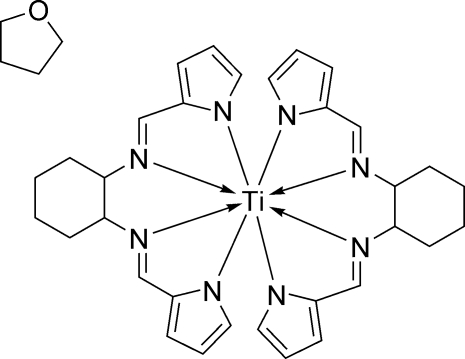


## Experimental

### 

#### Crystal data

[Ti(C_16_H_18_N_4_)_2_]·C_4_H_8_O
*M* *_r_* = 652.69
Monoclinic, 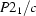

*a* = 15.7746 (11) Å
*b* = 8.7372 (6) Å
*c* = 23.5824 (16) Å
β = 90.214 (1)°
*V* = 3250.2 (4) Å^3^
*Z* = 4
Mo *K*α radiation
μ = 0.31 mm^−1^
*T* = 293 (2) K
0.44 × 0.34 × 0.24 mm

#### Data collection

Bruker SMART CCD area-detector diffractometer
Absorption correction: multi-scan (*SADABS*; Sheldrick, 1996 ▶) *T*_min_ = 0.877, *T*_max_ = 0.931
23461 measured reflections
6052 independent reflections
4955 reflections with *I* > 2σ(*I*)
*R*_int_ = 0.026

#### Refinement

*R*[*F* ^2^ > 2σ(*F* ^2^)] = 0.037
*wR*(*F* ^2^) = 0.101
*S* = 1.03
6052 reflections
415 parameters
H-atom parameters constrained
Δρ_max_ = 0.30 e Å^−3^
Δρ_min_ = −0.29 e Å^−3^

### 

Data collection: *SMART* (Siemens, 1996 ▶); cell refinement: *SAINT* (Siemens, 1996 ▶); data reduction: *SAINT*; program(s) used to solve structure: *SHELXTL* (Sheldrick, 2008 ▶); program(s) used to refine structure: *SHELXTL*; molecular graphics: *SHELXTL*; software used to prepare material for publication: *SHELXTL*.

## Supplementary Material

Crystal structure: contains datablocks I, global. DOI: 10.1107/S1600536808002870/xu2396sup1.cif

Structure factors: contains datablocks I. DOI: 10.1107/S1600536808002870/xu2396Isup2.hkl

Additional supplementary materials:  crystallographic information; 3D view; checkCIF report

## Figures and Tables

**Table 1:** Selected bond lengths (Å)

Ti1—N1	2.1289 (16)
Ti1—N2	2.2283 (15)
Ti1—N3	2.2344 (16)
Ti1—N4	2.2706 (16)
Ti1—N5	2.2929 (16)
Ti1—N6	2.2234 (15)
Ti1—N7	2.2593 (16)
Ti1—N8	2.1647 (16)

**Fig. 1.**
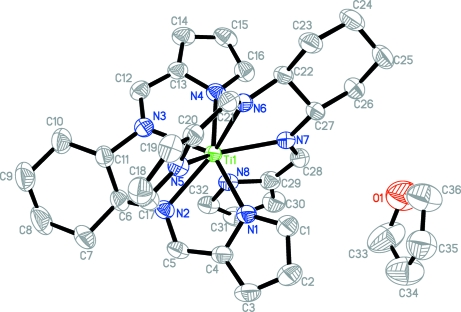
The molecular structure of the title compound. Displacement ellipsoids are drawn at the 50% probability level. H atoms have been omitted for clarity.

## supplementary crystallographic information

### Comment

It is well known that titanium metal reacts with N-donor ligands to form lots of complexes (Li *et al.*, 2002; Gardner *et al.*, 2001). Complexes synthesized from titanium and pyrrol-2-yl Schiff base ligands are important in coordination chemistry and catalysis (Han *et al.*, 2007). The bisligand coordinated titanium complexes are rare (Li *et al.*, 2002). Herein we report the synthesis and crystal structure of the title titanium complex.

The compound is an mononuclear titanium(IV) complex (Fig. 1). The Ti^IV^ ion is coordinated by eight N atoms from two ligands, forming a distorted square-antiprism geometry (Table 1). The lattice THF molecule assumes an envelope conformation.

### Experimental

1. Synthesis of the ligand

To an ethanol solution (15 ml) of pyrrole-2-carbaldehyde (3.804 g, 4 mmol) was added a solution of cyclohexane-1,2-diamine (2.284 g, 2 mmol) in ethanol (10 ml). The resulting mixture was stirred at room temperature for 1 h, and then a few drops of acetic acid were added to yield a white muddle mixture. The solid was collected by filtration, washed with cold ethanol and dried under vacuum to get the crude products, finally was purified by recrystallization from ethanol.

2. Synthesis of the complex

To a solution of Ti(NMe_2_)_4_ (0.448 g, 2 mmol) in THF (5 ml) at -78 °C was added a solution of the above ligand (1.0734 g, 4 mmol) in THF dropwise. The resulting mixture was stirred overnight at room temperature to yield a black solution. The solvent was removed from reduced pressure and a black solid was obtained. The black solid was washed with hexane (3 × 15 ml) and black single crystals were obtained *via* recrystallization from THF/hexane mixture at room temperature after 3 weeks.

### Refinement

H atoms were placed in calculated positions with C—H = 0.93 (aromatic) or 0.97 Å (methylene) and refined in riding mode, *U*_iso_(H) = 1.2*U*_eq_(C).

### Crystal data

**Table:**

[Ti(C_16_H_18_N_4_)_2_]·C_4_H_8_O	*F*(000) = 1384
*M**_r_* = 652.69	*D*_x_ = 1.334 Mg m^−^^3^
Monoclinic, *P*2_1_/*c*	Mo *K*α radiation, λ = 0.71073 Å
Hall symbol: -P 2ybc	Cell parameters from 7300 reflections
*a* = 15.7746 (11) Å	θ = 2.5–27.3°
*b* = 8.7372 (6) Å	µ = 0.31 mm^−^^1^
*c* = 23.5824 (16) Å	*T* = 293 K
β = 90.214 (1)°	Block, black
*V* = 3250.2 (4) Å^3^	0.44 × 0.34 × 0.24 mm
*Z* = 4	

### Data collection

**Table:**

Bruker SMART CCD area-detector diffractometer	6052 independent reflections
Radiation source: fine-focus sealed tube	4955 reflections with *I* > 2σ(*I*)
graphite	*R*_int_ = 0.026
φ and ω scans	θ_max_ = 25.5°, θ_min_ = 2.5°
Absorption correction: multi-scan (*SADABS*; Sheldrick, 1996)	*h* = −18→19
*T*_min_ = 0.877, *T*_max_ = 0.931	*k* = −10→10
23461 measured reflections	*l* = −28→28

### Refinement

**Table:**

Refinement on *F*^2^	Primary atom site location: structure-invariant direct methods
Least-squares matrix: full	Secondary atom site location: difference Fourier map
*R*[*F*^2^ > 2σ(*F*^2^)] = 0.037	Hydrogen site location: inferred from neighbouring sites
*wR*(*F*^2^) = 0.101	H-atom parameters constrained
*S* = 1.03	*w* = 1/[σ^2^(*F*_o_^2^) + (0.0465*P*)^2^ + 1.5415*P*] where *P* = (*F*_o_^2^ + 2*F*_c_^2^)/3
6052 reflections	(Δ/σ)_max_ = 0.001
415 parameters	Δρ_max_ = 0.31 e Å^−^^3^
0 restraints	Δρ_min_ = −0.29 e Å^−^^3^

### Special details

**Table:**

Geometry. All e.s.d.'s (except the e.s.d. in the dihedral angle between two l.s. planes) are estimated using the full covariance matrix. The cell e.s.d.'s are taken into account individually in the estimation of e.s.d.'s in distances, angles and torsion angles; correlations between e.s.d.'s in cell parameters are only used when they are defined by crystal symmetry. An approximate (isotropic) treatment of cell e.s.d.'s is used for estimating e.s.d.'s involving l.s. planes.
Refinement. Refinement of *F*^2^ against ALL reflections. The weighted *R*-factor *wR* and goodness of fit *S* are based on *F*^2^, conventional *R*-factors *R* are based on *F*, with *F* set to zero for negative *F*^2^. The threshold expression of *F*^2^ > σ(*F*^2^) is used only for calculating *R*-factors(gt) *etc*. and is not relevant to the choice of reflections for refinement. *R*-factors based on *F*^2^ are statistically about twice as large as those based on *F*, and *R*- factors based on ALL data will be even larger.

### Fractional atomic coordinates and isotropic or equivalent isotropic displacement parameters (Å^2^)

**Table:**

	*x*	*y*	*z*	*U*_iso_*/*U*_eq_	
Ti1	0.22892 (2)	0.45676 (4)	0.457549 (14)	0.02822 (10)	
O1	0.30387 (15)	0.7799 (3)	0.23814 (11)	0.0957 (7)	
N1	0.29834 (10)	0.66509 (18)	0.45179 (7)	0.0338 (4)	
N2	0.33519 (10)	0.43876 (18)	0.51971 (7)	0.0332 (4)	
N3	0.20532 (10)	0.26836 (18)	0.51959 (7)	0.0361 (4)	
N4	0.16564 (10)	0.25700 (18)	0.41265 (7)	0.0349 (4)	
N5	0.18761 (10)	0.58944 (18)	0.53665 (7)	0.0344 (4)	
N6	0.09990 (9)	0.55213 (17)	0.44335 (7)	0.0322 (3)	
N7	0.20985 (10)	0.52880 (18)	0.36638 (7)	0.0331 (4)	
N8	0.33591 (10)	0.37197 (18)	0.40968 (7)	0.0349 (4)	
C1	0.28983 (14)	0.8006 (2)	0.42528 (10)	0.0464 (5)	
H1	0.2471	0.8240	0.3994	0.056*	
C2	0.35369 (17)	0.9008 (3)	0.44180 (12)	0.0625 (7)	
H2	0.3608	1.0011	0.4294	0.075*	
C3	0.40447 (15)	0.8236 (3)	0.47998 (11)	0.0547 (6)	
H3	0.4526	0.8611	0.4982	0.066*	
C4	0.36938 (12)	0.6782 (2)	0.48590 (9)	0.0380 (5)	
C5	0.38696 (12)	0.5526 (2)	0.52143 (9)	0.0388 (5)	
H5	0.4341	0.5519	0.5452	0.047*	
C6	0.34152 (13)	0.3068 (2)	0.55825 (8)	0.0392 (5)	
H6	0.3637	0.2208	0.5362	0.047*	
C7	0.39832 (15)	0.3249 (3)	0.61032 (10)	0.0537 (6)	
H7A	0.3797	0.4118	0.6326	0.064*	
H7B	0.4563	0.3432	0.5986	0.064*	
C8	0.39389 (18)	0.1792 (3)	0.64598 (11)	0.0706 (8)	
H8A	0.4199	0.0958	0.6252	0.085*	
H8B	0.4261	0.1941	0.6806	0.085*	
C9	0.30358 (19)	0.1351 (3)	0.66102 (11)	0.0694 (8)	
H9A	0.2797	0.2124	0.6858	0.083*	
H9B	0.3041	0.0387	0.6815	0.083*	
C10	0.24785 (17)	0.1193 (3)	0.60826 (10)	0.0559 (6)	
H10A	0.2680	0.0350	0.5851	0.067*	
H10B	0.1899	0.0974	0.6194	0.067*	
C11	0.25059 (13)	0.2674 (2)	0.57418 (8)	0.0392 (5)	
H11	0.2281	0.3503	0.5978	0.047*	
C12	0.16251 (13)	0.1497 (2)	0.50368 (9)	0.0403 (5)	
H12	0.1490	0.0725	0.5292	0.048*	
C13	0.13727 (12)	0.1411 (2)	0.44710 (9)	0.0365 (4)	
C14	0.10000 (14)	0.0242 (2)	0.41541 (10)	0.0460 (5)	
H14	0.0760	−0.0656	0.4292	0.055*	
C15	0.10594 (14)	0.0686 (2)	0.35952 (10)	0.0455 (5)	
H15	0.0867	0.0145	0.3280	0.055*	
C16	0.14637 (13)	0.2102 (2)	0.35930 (9)	0.0413 (5)	
H16	0.1586	0.2657	0.3267	0.050*	
C17	0.21998 (13)	0.6389 (2)	0.58649 (9)	0.0415 (5)	
H17	0.2737	0.6136	0.5999	0.050*	
C18	0.16340 (14)	0.7320 (2)	0.61535 (9)	0.0449 (5)	
H18	0.1721	0.7781	0.6504	0.054*	
C19	0.09170 (14)	0.7427 (2)	0.58181 (9)	0.0434 (5)	
H19	0.0426	0.7976	0.5896	0.052*	
C20	0.10798 (12)	0.6543 (2)	0.53367 (8)	0.0343 (4)	
C21	0.06392 (12)	0.6318 (2)	0.48316 (8)	0.0354 (4)	
H21	0.0100	0.6728	0.4780	0.043*	
C22	0.06192 (12)	0.5472 (2)	0.38635 (8)	0.0343 (4)	
H22	0.0516	0.4396	0.3768	0.041*	
C23	−0.02049 (13)	0.6350 (2)	0.37604 (9)	0.0437 (5)	
H23A	−0.0126	0.7422	0.3854	0.052*	
H23B	−0.0650	0.5943	0.4000	0.052*	
C24	−0.04587 (14)	0.6190 (3)	0.31342 (10)	0.0534 (6)	
H24A	−0.0583	0.5125	0.3053	0.064*	
H24B	−0.0970	0.6779	0.3064	0.064*	
C25	0.02408 (15)	0.6744 (3)	0.27410 (10)	0.0558 (6)	
H25A	0.0329	0.7832	0.2798	0.067*	
H25B	0.0067	0.6589	0.2350	0.067*	
C26	0.10726 (14)	0.5891 (3)	0.28511 (9)	0.0480 (5)	
H26A	0.1005	0.4817	0.2758	0.058*	
H26B	0.1517	0.6310	0.2614	0.058*	
C27	0.13184 (12)	0.6060 (2)	0.34756 (8)	0.0359 (4)	
H27	0.1388	0.7155	0.3552	0.043*	
C28	0.27018 (13)	0.4974 (2)	0.33193 (9)	0.0406 (5)	
H28	0.2678	0.5281	0.2942	0.049*	
C29	0.34005 (13)	0.4148 (2)	0.35323 (9)	0.0406 (5)	
C30	0.41538 (14)	0.3658 (3)	0.32947 (10)	0.0524 (6)	
H30	0.4329	0.3808	0.2923	0.063*	
C31	0.45961 (14)	0.2901 (3)	0.37197 (10)	0.0508 (6)	
H31	0.5125	0.2439	0.3688	0.061*	
C32	0.40960 (12)	0.2968 (2)	0.42010 (9)	0.0409 (5)	
H32	0.4246	0.2551	0.4550	0.049*	
C33	0.3873 (2)	0.7946 (4)	0.2598 (2)	0.1107 (14)	
H33A	0.4279	0.7614	0.2315	0.133*	
H33B	0.3941	0.7309	0.2932	0.133*	
C34	0.4027 (2)	0.9553 (4)	0.27450 (18)	0.0978 (11)	
H34A	0.4349	1.0062	0.2450	0.117*	
H34B	0.4336	0.9633	0.3100	0.117*	
C35	0.31730 (19)	1.0227 (4)	0.27958 (14)	0.0783 (8)	
H35A	0.3013	1.0324	0.3191	0.094*	
H35B	0.3153	1.1231	0.2620	0.094*	
C36	0.2606 (2)	0.9156 (4)	0.24985 (16)	0.0888 (10)	
H36A	0.2117	0.8939	0.2733	0.107*	
H36B	0.2409	0.9614	0.2147	0.107*	

### Atomic displacement parameters (Å^2^)

**Table:**

	*U*^11^	*U*^22^	*U*^33^	*U*^12^	*U*^13^	*U*^23^
Ti1	0.02674 (18)	0.02732 (18)	0.03059 (18)	−0.00220 (13)	−0.00331 (13)	0.00056 (13)
O1	0.0962 (17)	0.0741 (15)	0.1167 (19)	0.0006 (13)	−0.0101 (14)	−0.0173 (13)
N1	0.0332 (8)	0.0305 (8)	0.0376 (9)	−0.0039 (7)	−0.0047 (7)	0.0007 (7)
N2	0.0300 (8)	0.0331 (8)	0.0365 (9)	0.0014 (7)	−0.0041 (7)	0.0004 (7)
N3	0.0382 (9)	0.0333 (9)	0.0367 (9)	−0.0034 (7)	−0.0034 (7)	0.0021 (7)
N4	0.0351 (9)	0.0300 (8)	0.0396 (9)	−0.0008 (7)	−0.0052 (7)	−0.0021 (7)
N5	0.0345 (9)	0.0325 (8)	0.0362 (9)	0.0014 (7)	−0.0009 (7)	−0.0013 (7)
N6	0.0292 (8)	0.0307 (8)	0.0366 (9)	−0.0022 (7)	−0.0031 (7)	0.0023 (7)
N7	0.0318 (8)	0.0339 (9)	0.0337 (8)	−0.0059 (7)	−0.0034 (7)	0.0014 (7)
N8	0.0305 (8)	0.0341 (9)	0.0401 (9)	−0.0026 (7)	−0.0017 (7)	−0.0028 (7)
C1	0.0491 (13)	0.0360 (11)	0.0541 (13)	−0.0057 (10)	−0.0117 (10)	0.0059 (10)
C2	0.0733 (17)	0.0354 (12)	0.0786 (18)	−0.0194 (12)	−0.0214 (14)	0.0123 (12)
C3	0.0509 (14)	0.0441 (13)	0.0689 (16)	−0.0183 (11)	−0.0164 (12)	0.0018 (11)
C4	0.0340 (10)	0.0366 (11)	0.0434 (11)	−0.0068 (8)	−0.0059 (9)	−0.0011 (9)
C5	0.0298 (10)	0.0434 (12)	0.0432 (11)	−0.0019 (9)	−0.0102 (8)	−0.0033 (9)
C6	0.0417 (11)	0.0375 (11)	0.0383 (11)	0.0063 (9)	−0.0074 (9)	0.0036 (9)
C7	0.0509 (14)	0.0614 (15)	0.0487 (13)	0.0039 (11)	−0.0171 (11)	0.0074 (11)
C8	0.082 (2)	0.0743 (18)	0.0558 (16)	0.0111 (15)	−0.0279 (14)	0.0159 (14)
C9	0.095 (2)	0.0637 (17)	0.0495 (15)	−0.0052 (15)	−0.0161 (14)	0.0214 (13)
C10	0.0734 (16)	0.0468 (13)	0.0472 (13)	−0.0067 (12)	−0.0074 (12)	0.0125 (11)
C11	0.0488 (12)	0.0352 (11)	0.0335 (10)	−0.0010 (9)	−0.0061 (9)	0.0030 (8)
C12	0.0412 (11)	0.0330 (10)	0.0467 (12)	−0.0059 (9)	−0.0004 (9)	0.0063 (9)
C13	0.0331 (10)	0.0291 (10)	0.0472 (12)	−0.0025 (8)	−0.0052 (8)	0.0024 (9)
C14	0.0437 (12)	0.0303 (11)	0.0638 (15)	−0.0054 (9)	−0.0097 (10)	−0.0020 (10)
C15	0.0465 (12)	0.0346 (11)	0.0553 (14)	0.0034 (9)	−0.0153 (10)	−0.0132 (10)
C16	0.0466 (12)	0.0368 (11)	0.0404 (11)	0.0019 (9)	−0.0059 (9)	−0.0039 (9)
C17	0.0437 (12)	0.0406 (11)	0.0400 (11)	0.0011 (9)	−0.0071 (9)	−0.0027 (9)
C18	0.0600 (14)	0.0370 (11)	0.0375 (11)	−0.0005 (10)	−0.0002 (10)	−0.0064 (9)
C19	0.0477 (12)	0.0379 (11)	0.0445 (12)	0.0075 (9)	0.0075 (10)	−0.0004 (9)
C20	0.0348 (10)	0.0312 (10)	0.0370 (11)	0.0014 (8)	0.0034 (8)	0.0029 (8)
C21	0.0295 (10)	0.0315 (10)	0.0453 (11)	0.0010 (8)	0.0014 (8)	0.0060 (9)
C22	0.0323 (10)	0.0319 (10)	0.0386 (10)	−0.0034 (8)	−0.0087 (8)	0.0021 (8)
C23	0.0357 (11)	0.0390 (11)	0.0562 (13)	−0.0004 (9)	−0.0115 (9)	0.0027 (10)
C24	0.0452 (13)	0.0529 (14)	0.0620 (15)	−0.0017 (11)	−0.0251 (11)	0.0014 (12)
C25	0.0612 (15)	0.0568 (14)	0.0494 (14)	−0.0063 (12)	−0.0279 (12)	0.0075 (11)
C26	0.0507 (13)	0.0542 (13)	0.0391 (12)	−0.0089 (11)	−0.0119 (10)	0.0047 (10)
C27	0.0352 (10)	0.0351 (10)	0.0375 (11)	−0.0047 (8)	−0.0104 (8)	0.0035 (8)
C28	0.0415 (11)	0.0491 (12)	0.0312 (10)	−0.0082 (9)	−0.0009 (9)	0.0017 (9)
C29	0.0365 (11)	0.0474 (12)	0.0378 (11)	−0.0039 (9)	0.0029 (8)	−0.0028 (9)
C30	0.0428 (12)	0.0679 (16)	0.0465 (13)	−0.0025 (11)	0.0108 (10)	−0.0069 (12)
C31	0.0330 (11)	0.0575 (14)	0.0621 (15)	0.0040 (10)	0.0040 (10)	−0.0146 (12)
C32	0.0337 (11)	0.0385 (11)	0.0507 (12)	0.0005 (9)	−0.0040 (9)	−0.0052 (9)
C33	0.062 (2)	0.074 (2)	0.197 (4)	0.0054 (17)	0.030 (2)	0.012 (3)
C34	0.066 (2)	0.096 (3)	0.132 (3)	0.0034 (18)	0.001 (2)	−0.024 (2)
C35	0.076 (2)	0.073 (2)	0.086 (2)	0.0042 (16)	−0.0047 (16)	−0.0032 (16)
C36	0.070 (2)	0.087 (2)	0.110 (3)	0.0045 (17)	−0.0120 (18)	−0.020 (2)

### Geometric parameters (Å, °)

**Table:**

Ti1—N1	2.1289 (16)	C12—H12	0.9300
Ti1—N2	2.2283 (15)	C13—C14	1.394 (3)
Ti1—N3	2.2344 (16)	C14—C15	1.378 (3)
Ti1—N4	2.2706 (16)	C14—H14	0.9300
Ti1—N5	2.2929 (16)	C15—C16	1.392 (3)
Ti1—N6	2.2234 (15)	C15—H15	0.9300
Ti1—N7	2.2593 (16)	C16—H16	0.9300
Ti1—N8	2.1647 (16)	C17—C18	1.388 (3)
O1—C36	1.396 (4)	C17—H17	0.9300
O1—C33	1.415 (4)	C18—C19	1.381 (3)
N1—C1	1.345 (3)	C18—H18	0.9300
N1—C4	1.382 (2)	C19—C20	1.398 (3)
N2—C5	1.288 (2)	C19—H19	0.9300
N2—C6	1.471 (2)	C20—C21	1.391 (3)
N3—C12	1.292 (2)	C21—H21	0.9300
N3—C11	1.470 (2)	C22—C27	1.525 (3)
N4—C16	1.356 (3)	C22—C23	1.528 (3)
N4—C13	1.374 (2)	C22—H22	0.9800
N5—C17	1.351 (3)	C23—C24	1.535 (3)
N5—C20	1.380 (2)	C23—H23A	0.9700
N6—C21	1.301 (2)	C23—H23B	0.9700
N6—C22	1.470 (2)	C24—C25	1.523 (3)
N7—C28	1.283 (3)	C24—H24A	0.9700
N7—C27	1.470 (2)	C24—H24B	0.9700
N8—C32	1.357 (2)	C25—C26	1.530 (3)
N8—C29	1.385 (3)	C25—H25A	0.9700
C1—C2	1.390 (3)	C25—H25B	0.9700
C1—H1	0.9300	C26—C27	1.529 (3)
C2—C3	1.379 (3)	C26—H26A	0.9700
C2—H2	0.9300	C26—H26B	0.9700
C3—C4	1.393 (3)	C27—H27	0.9800
C3—H3	0.9300	C28—C29	1.408 (3)
C4—C5	1.407 (3)	C28—H28	0.9300
C5—H5	0.9300	C29—C30	1.384 (3)
C6—C11	1.523 (3)	C30—C31	1.387 (3)
C6—C7	1.525 (3)	C30—H30	0.9300
C6—H6	0.9800	C31—C32	1.386 (3)
C7—C8	1.528 (4)	C31—H31	0.9300
C7—H7A	0.9700	C32—H32	0.9300
C7—H7B	0.9700	C33—C34	1.466 (5)
C8—C9	1.519 (4)	C33—H33A	0.9700
C8—H8A	0.9700	C33—H33B	0.9700
C8—H8B	0.9700	C34—C35	1.476 (4)
C9—C10	1.527 (3)	C34—H34A	0.9700
C9—H9A	0.9700	C34—H34B	0.9700
C9—H9B	0.9700	C35—C36	1.470 (4)
C10—C11	1.525 (3)	C35—H35A	0.9700
C10—H10A	0.9700	C35—H35B	0.9700
C10—H10B	0.9700	C36—H36A	0.9700
C11—H11	0.9800	C36—H36B	0.9700
C12—C13	1.393 (3)		
			
N1—Ti1—N8	81.79 (6)	N3—C12—H12	121.0
N1—Ti1—N6	98.09 (6)	C13—C12—H12	121.0
N8—Ti1—N6	139.89 (6)	N4—C13—C12	115.70 (17)
N1—Ti1—N2	73.52 (6)	N4—C13—C14	111.15 (18)
N8—Ti1—N2	74.50 (6)	C12—C13—C14	132.18 (19)
N6—Ti1—N2	144.24 (6)	C15—C14—C13	106.04 (19)
N1—Ti1—N3	139.35 (6)	C15—C14—H14	127.0
N8—Ti1—N3	102.79 (6)	C13—C14—H14	127.0
N6—Ti1—N3	102.71 (6)	C14—C15—C16	106.66 (19)
N2—Ti1—N3	69.15 (6)	C14—C15—H15	126.7
N1—Ti1—N7	76.57 (6)	C16—C15—H15	126.7
N8—Ti1—N7	72.56 (6)	N4—C16—C15	111.45 (19)
N6—Ti1—N7	68.51 (6)	N4—C16—H16	124.3
N2—Ti1—N7	137.97 (6)	C15—C16—H16	124.3
N3—Ti1—N7	143.75 (6)	N5—C17—C18	111.87 (18)
N1—Ti1—N4	148.52 (6)	N5—C17—H17	124.1
N8—Ti1—N4	80.56 (6)	C18—C17—H17	124.1
N6—Ti1—N4	79.47 (6)	C19—C18—C17	106.61 (19)
N2—Ti1—N4	125.51 (6)	C19—C18—H18	126.7
N3—Ti1—N4	70.43 (6)	C17—C18—H18	126.7
N7—Ti1—N4	73.36 (6)	C18—C19—C20	105.96 (18)
N1—Ti1—N5	76.55 (6)	C18—C19—H19	127.0
N8—Ti1—N5	145.27 (6)	C20—C19—H19	127.0
N6—Ti1—N5	70.75 (6)	N5—C20—C21	115.96 (17)
N2—Ti1—N5	73.49 (6)	N5—C20—C19	110.83 (17)
N3—Ti1—N5	77.95 (6)	C21—C20—C19	132.91 (19)
N7—Ti1—N5	126.59 (6)	N6—C21—C20	118.40 (17)
N4—Ti1—N5	129.96 (6)	N6—C21—H21	120.8
C36—O1—C33	107.8 (3)	C20—C21—H21	120.8
C1—N1—C4	106.07 (16)	N6—C22—C27	104.20 (14)
C1—N1—Ti1	137.02 (14)	N6—C22—C23	118.31 (17)
C4—N1—Ti1	116.75 (12)	C27—C22—C23	110.62 (16)
C5—N2—C6	122.99 (16)	N6—C22—H22	107.8
C5—N2—Ti1	116.20 (13)	C27—C22—H22	107.8
C6—N2—Ti1	120.72 (12)	C23—C22—H22	107.8
C12—N3—C11	120.11 (17)	C22—C23—C24	109.02 (18)
C12—N3—Ti1	119.28 (13)	C22—C23—H23A	109.9
C11—N3—Ti1	119.73 (12)	C24—C23—H23A	109.9
C16—N4—C13	104.70 (16)	C22—C23—H23B	109.9
C16—N4—Ti1	139.54 (14)	C24—C23—H23B	109.9
C13—N4—Ti1	115.75 (12)	H23A—C23—H23B	108.3
C17—N5—C20	104.73 (16)	C25—C24—C23	111.70 (18)
C17—N5—Ti1	139.64 (13)	C25—C24—H24A	109.3
C20—N5—Ti1	115.32 (12)	C23—C24—H24A	109.3
C21—N6—C22	119.87 (16)	C25—C24—H24B	109.3
C21—N6—Ti1	119.57 (13)	C23—C24—H24B	109.3
C22—N6—Ti1	119.76 (12)	H24A—C24—H24B	107.9
C28—N7—C27	121.93 (17)	C24—C25—C26	111.37 (19)
C28—N7—Ti1	116.52 (13)	C24—C25—H25A	109.4
C27—N7—Ti1	121.55 (12)	C26—C25—H25A	109.4
C32—N8—C29	105.17 (16)	C24—C25—H25B	109.4
C32—N8—Ti1	137.76 (14)	C26—C25—H25B	109.4
C29—N8—Ti1	116.64 (13)	H25A—C25—H25B	108.0
N1—C1—C2	110.69 (19)	C27—C26—C25	109.32 (19)
N1—C1—H1	124.7	C27—C26—H26A	109.8
C2—C1—H1	124.7	C25—C26—H26A	109.8
C3—C2—C1	107.1 (2)	C27—C26—H26B	109.8
C3—C2—H2	126.5	C25—C26—H26B	109.8
C1—C2—H2	126.5	H26A—C26—H26B	108.3
C2—C3—C4	106.35 (19)	N7—C27—C22	105.71 (15)
C2—C3—H3	126.8	N7—C27—C26	117.10 (17)
C4—C3—H3	126.8	C22—C27—C26	111.35 (16)
N1—C4—C3	109.82 (18)	N7—C27—H27	107.4
N1—C4—C5	116.07 (17)	C22—C27—H27	107.4
C3—C4—C5	133.88 (19)	C26—C27—H27	107.4
N2—C5—C4	117.40 (17)	N7—C28—C29	117.71 (19)
N2—C5—H5	121.3	N7—C28—H28	121.1
C4—C5—H5	121.3	C29—C28—H28	121.1
N2—C6—C11	105.52 (15)	C30—C29—N8	110.45 (19)
N2—C6—C7	117.03 (17)	C30—C29—C28	133.3 (2)
C11—C6—C7	112.07 (18)	N8—C29—C28	116.22 (18)
N2—C6—H6	107.3	C29—C30—C31	106.6 (2)
C11—C6—H6	107.3	C29—C30—H30	126.7
C7—C6—H6	107.3	C31—C30—H30	126.7
C6—C7—C8	109.2 (2)	C32—C31—C30	106.59 (19)
C6—C7—H7A	109.8	C32—C31—H31	126.7
C8—C7—H7A	109.8	C30—C31—H31	126.7
C6—C7—H7B	109.8	N8—C32—C31	111.2 (2)
C8—C7—H7B	109.8	N8—C32—H32	124.4
H7A—C7—H7B	108.3	C31—C32—H32	124.4
C9—C8—C7	112.6 (2)	O1—C33—C34	109.0 (3)
C9—C8—H8A	109.1	O1—C33—H33A	109.9
C7—C8—H8A	109.1	C34—C33—H33A	109.9
C9—C8—H8B	109.1	O1—C33—H33B	109.9
C7—C8—H8B	109.1	C34—C33—H33B	109.9
H8A—C8—H8B	107.8	H33A—C33—H33B	108.3
C8—C9—C10	111.7 (2)	C33—C34—C35	104.5 (3)
C8—C9—H9A	109.3	C33—C34—H34A	110.9
C10—C9—H9A	109.3	C35—C34—H34A	110.9
C8—C9—H9B	109.3	C33—C34—H34B	110.9
C10—C9—H9B	109.3	C35—C34—H34B	110.9
H9A—C9—H9B	107.9	H34A—C34—H34B	108.9
C11—C10—C9	109.6 (2)	C36—C35—C34	105.1 (3)
C11—C10—H10A	109.8	C36—C35—H35A	110.7
C9—C10—H10A	109.8	C34—C35—H35A	110.7
C11—C10—H10B	109.8	C36—C35—H35B	110.7
C9—C10—H10B	109.8	C34—C35—H35B	110.7
H10A—C10—H10B	108.2	H35A—C35—H35B	108.8
N3—C11—C6	103.74 (15)	O1—C36—C35	109.8 (3)
N3—C11—C10	116.86 (17)	O1—C36—H36A	109.7
C6—C11—C10	110.52 (18)	C35—C36—H36A	109.7
N3—C11—H11	108.5	O1—C36—H36B	109.7
C6—C11—H11	108.5	C35—C36—H36B	109.7
C10—C11—H11	108.5	H36A—C36—H36B	108.2
N3—C12—C13	117.96 (18)		
			
N8—Ti1—N1—C1	110.8 (2)	N6—Ti1—N8—C29	19.56 (18)
N6—Ti1—N1—C1	−28.6 (2)	N2—Ti1—N8—C29	−148.36 (15)
N2—Ti1—N1—C1	−173.0 (2)	N3—Ti1—N8—C29	147.86 (14)
N3—Ti1—N1—C1	−149.07 (19)	N7—Ti1—N8—C29	5.16 (13)
N7—Ti1—N1—C1	36.9 (2)	N4—Ti1—N8—C29	80.56 (14)
N4—Ti1—N1—C1	54.4 (3)	N5—Ti1—N8—C29	−124.91 (14)
N5—Ti1—N1—C1	−96.5 (2)	C4—N1—C1—C2	−0.3 (3)
N8—Ti1—N1—C4	−74.52 (14)	Ti1—N1—C1—C2	174.77 (18)
N6—Ti1—N1—C4	146.02 (14)	N1—C1—C2—C3	0.4 (3)
N2—Ti1—N1—C4	1.66 (14)	C1—C2—C3—C4	−0.4 (3)
N3—Ti1—N1—C4	25.58 (19)	C1—N1—C4—C3	0.0 (2)
N7—Ti1—N1—C4	−148.45 (15)	Ti1—N1—C4—C3	−176.24 (15)
N4—Ti1—N1—C4	−130.94 (14)	C1—N1—C4—C5	175.17 (19)
N5—Ti1—N1—C4	78.13 (14)	Ti1—N1—C4—C5	−1.0 (2)
N1—Ti1—N2—C5	−2.26 (14)	C2—C3—C4—N1	0.3 (3)
N8—Ti1—N2—C5	83.58 (15)	C2—C3—C4—C5	−173.7 (3)
N6—Ti1—N2—C5	−83.07 (17)	C6—N2—C5—C4	−174.16 (18)
N3—Ti1—N2—C5	−165.84 (16)	Ti1—N2—C5—C4	2.5 (2)
N7—Ti1—N2—C5	44.13 (18)	N1—C4—C5—N2	−1.0 (3)
N4—Ti1—N2—C5	149.57 (14)	C3—C4—C5—N2	172.7 (2)
N5—Ti1—N2—C5	−82.74 (15)	C5—N2—C6—C11	139.94 (19)
N1—Ti1—N2—C6	174.48 (15)	Ti1—N2—C6—C11	−36.56 (19)
N8—Ti1—N2—C6	−99.69 (14)	C5—N2—C6—C7	14.5 (3)
N6—Ti1—N2—C6	93.66 (16)	Ti1—N2—C6—C7	−161.97 (15)
N3—Ti1—N2—C6	10.89 (13)	N2—C6—C7—C8	177.8 (2)
N7—Ti1—N2—C6	−139.14 (13)	C11—C6—C7—C8	55.7 (3)
N4—Ti1—N2—C6	−33.70 (16)	C6—C7—C8—C9	−53.9 (3)
N5—Ti1—N2—C6	93.99 (14)	C7—C8—C9—C10	55.3 (3)
N1—Ti1—N3—C12	−173.70 (14)	C8—C9—C10—C11	−55.9 (3)
N8—Ti1—N3—C12	−81.44 (16)	C12—N3—C11—C6	125.00 (19)
N6—Ti1—N3—C12	67.34 (16)	Ti1—N3—C11—C6	−44.17 (19)
N2—Ti1—N3—C12	−149.12 (17)	C12—N3—C11—C10	3.1 (3)
N7—Ti1—N3—C12	−3.6 (2)	Ti1—N3—C11—C10	−166.06 (16)
N4—Ti1—N3—C12	−6.46 (15)	N2—C6—C11—N3	46.80 (19)
N5—Ti1—N3—C12	134.15 (16)	C7—C6—C11—N3	175.23 (17)
N1—Ti1—N3—C11	−4.44 (19)	N2—C6—C11—C10	172.82 (17)
N8—Ti1—N3—C11	87.83 (15)	C7—C6—C11—C10	−58.7 (2)
N6—Ti1—N3—C11	−123.40 (14)	C9—C10—C11—N3	175.6 (2)
N2—Ti1—N3—C11	20.14 (14)	C9—C10—C11—C6	57.3 (3)
N7—Ti1—N3—C11	165.71 (13)	C11—N3—C12—C13	−165.45 (18)
N4—Ti1—N3—C11	162.80 (15)	Ti1—N3—C12—C13	3.8 (2)
N5—Ti1—N3—C11	−56.59 (14)	C16—N4—C13—C12	169.51 (18)
N1—Ti1—N4—C16	−6.4 (3)	Ti1—N4—C13—C12	−9.6 (2)
N8—Ti1—N4—C16	−63.1 (2)	C16—N4—C13—C14	−0.6 (2)
N6—Ti1—N4—C16	81.9 (2)	Ti1—N4—C13—C14	−179.69 (13)
N2—Ti1—N4—C16	−126.3 (2)	N3—C12—C13—N4	4.0 (3)
N3—Ti1—N4—C16	−170.4 (2)	N3—C12—C13—C14	171.5 (2)
N7—Ti1—N4—C16	11.4 (2)	N4—C13—C14—C15	0.4 (2)
N5—Ti1—N4—C16	135.55 (19)	C12—C13—C14—C15	−167.5 (2)
N1—Ti1—N4—C13	172.28 (13)	C13—C14—C15—C16	0.0 (2)
N8—Ti1—N4—C13	115.57 (14)	C13—N4—C16—C15	0.6 (2)
N6—Ti1—N4—C13	−99.41 (14)	Ti1—N4—C16—C15	179.32 (15)
N2—Ti1—N4—C13	52.40 (15)	C14—C15—C16—N4	−0.3 (2)
N3—Ti1—N4—C13	8.27 (13)	C20—N5—C17—C18	0.4 (2)
N7—Ti1—N4—C13	−169.94 (14)	Ti1—N5—C17—C18	173.18 (15)
N5—Ti1—N4—C13	−45.79 (16)	N5—C17—C18—C19	−0.4 (3)
N1—Ti1—N5—C17	−68.2 (2)	C17—C18—C19—C20	0.2 (2)
N8—Ti1—N5—C17	−15.3 (3)	C17—N5—C20—C21	174.29 (17)
N6—Ti1—N5—C17	−171.9 (2)	Ti1—N5—C20—C21	−0.6 (2)
N2—Ti1—N5—C17	8.3 (2)	C17—N5—C20—C19	−0.2 (2)
N3—Ti1—N5—C17	79.8 (2)	Ti1—N5—C20—C19	−175.08 (13)
N7—Ti1—N5—C17	−129.9 (2)	C18—C19—C20—N5	0.0 (2)
N4—Ti1—N5—C17	131.1 (2)	C18—C19—C20—C21	−173.3 (2)
N1—Ti1—N5—C20	104.06 (14)	C22—N6—C21—C20	−169.89 (16)
N8—Ti1—N5—C20	157.00 (12)	Ti1—N6—C21—C20	−0.1 (2)
N6—Ti1—N5—C20	0.37 (12)	N5—C20—C21—N6	0.5 (3)
N2—Ti1—N5—C20	−179.43 (14)	C19—C20—C21—N6	173.5 (2)
N3—Ti1—N5—C20	−107.87 (14)	C21—N6—C22—C27	122.29 (18)
N7—Ti1—N5—C20	42.42 (15)	Ti1—N6—C22—C27	−47.47 (17)
N4—Ti1—N5—C20	−56.60 (15)	C21—N6—C22—C23	−1.0 (3)
N1—Ti1—N6—C21	−72.77 (14)	Ti1—N6—C22—C23	−170.76 (13)
N8—Ti1—N6—C21	−159.60 (13)	N6—C22—C23—C24	177.60 (17)
N2—Ti1—N6—C21	0.20 (19)	C27—C22—C23—C24	57.6 (2)
N3—Ti1—N6—C21	72.08 (14)	C22—C23—C24—C25	−56.7 (2)
N7—Ti1—N6—C21	−144.83 (15)	C23—C24—C25—C26	56.8 (3)
N4—Ti1—N6—C21	139.05 (15)	C24—C25—C26—C27	−55.7 (2)
N5—Ti1—N6—C21	−0.14 (13)	C28—N7—C27—C22	149.55 (18)
N1—Ti1—N6—C22	97.01 (13)	Ti1—N7—C27—C22	−29.71 (19)
N8—Ti1—N6—C22	10.18 (17)	C28—N7—C27—C26	24.9 (3)
N2—Ti1—N6—C22	169.99 (12)	Ti1—N7—C27—C26	−154.37 (14)
N3—Ti1—N6—C22	−118.14 (13)	N6—C22—C27—N7	44.41 (18)
N7—Ti1—N6—C22	24.95 (12)	C23—C22—C27—N7	172.57 (16)
N4—Ti1—N6—C22	−51.17 (13)	N6—C22—C27—C26	172.58 (16)
N5—Ti1—N6—C22	169.65 (14)	C23—C22—C27—C26	−59.3 (2)
N1—Ti1—N7—C28	80.97 (15)	C25—C26—C27—N7	178.97 (17)
N8—Ti1—N7—C28	−4.50 (14)	C25—C26—C27—C22	57.2 (2)
N6—Ti1—N7—C28	−174.59 (16)	C27—N7—C28—C29	−176.14 (18)
N2—Ti1—N7—C28	35.43 (18)	Ti1—N7—C28—C29	3.2 (2)
N3—Ti1—N7—C28	−92.45 (16)	C32—N8—C29—C30	0.2 (2)
N4—Ti1—N7—C28	−89.60 (15)	Ti1—N8—C29—C30	174.01 (15)
N5—Ti1—N7—C28	142.60 (14)	C32—N8—C29—C28	−179.39 (18)
N1—Ti1—N7—C27	−99.73 (14)	Ti1—N8—C29—C28	−5.6 (2)
N8—Ti1—N7—C27	174.80 (14)	N7—C28—C29—C30	−178.0 (2)
N6—Ti1—N7—C27	4.71 (13)	N7—C28—C29—N8	1.5 (3)
N2—Ti1—N7—C27	−145.27 (13)	N8—C29—C30—C31	0.1 (3)
N3—Ti1—N7—C27	86.85 (16)	C28—C29—C30—C31	179.6 (2)
N4—Ti1—N7—C27	89.70 (14)	C29—C30—C31—C32	−0.3 (3)
N5—Ti1—N7—C27	−38.10 (16)	C29—N8—C32—C31	−0.4 (2)
N1—Ti1—N8—C32	97.85 (19)	Ti1—N8—C32—C31	−172.18 (15)
N6—Ti1—N8—C32	−169.32 (17)	C30—C31—C32—N8	0.5 (3)
N2—Ti1—N8—C32	22.76 (19)	C36—O1—C33—C34	−12.5 (5)
N3—Ti1—N8—C32	−41.0 (2)	O1—C33—C34—C35	19.3 (5)
N7—Ti1—N8—C32	176.3 (2)	C33—C34—C35—C36	−18.2 (4)
N4—Ti1—N8—C32	−108.3 (2)	C33—O1—C36—C35	0.4 (4)
N5—Ti1—N8—C32	46.2 (2)	C34—C35—C36—O1	11.5 (4)
N1—Ti1—N8—C29	−73.27 (14)		
